# Syntax, action, comparative cognitive science, and Darwinian thinking

**DOI:** 10.3389/fpsyg.2014.00627

**Published:** 2014-06-27

**Authors:** Cedric A. Boeckx, Koji Fujita

**Affiliations:** ^1^Catalan Institute for Research and Advanced Studies (ICREA)Barcelona, Spain; ^2^Department of General Linguistics, Universitat de BarcelonaBarcelona, Spain; ^3^Department of Human Coexistence, Graduate School of Human and Environmental Studies, Kyoto UniversityKyoto, Japan

**Keywords:** syntax, action grammar, modularity, evolution, descent with modification, comparative cognition

In a recent exchange, Moro (2014) and Pulvermüller (2014) re-open a long-standing debate in the language sciences. Ever since the 1975 Royaumont encounter that set the agenda for linguistics and the classical cognitive sciences, generative linguists, whose ultimate goal we take to be to shed light on the biological basis for language, have rejected any attempt of a rapprochement between natural language syntax and action grammar (motor planning), even if the hierarchical structure of plans is well established in the literature (see already Miller et al., 1960). The parallelism between syntax and action grammar has enjoyed a new lease of life recently (Jackendoff, 2007; Fujita, 2009; Pulvermüller, 2010; Stout, 2010; Arbib, 2012; Knott, 2012), with neuroscientists like Pulvermüller ready to reap the fruits, but Moro reiterates the standard generative stance that the parallelism is at best a metaphor.

Moro usefully lays out two classic arguments against a deep relation between syntax and action: (i) the atomic units these systems manipulate are very different, and (ii) the locality conditions or constraints characteristic of syntax are not found in the domain of action.

We think Moro's arguments fail, for reasons that are worth highlighting, for both sides of the debate: First, the very same arguments Moro puts forth could be raised to argue against a relationship between syntax and other human capacities such as mathematics or music, although here generative linguists have in fact been known to promote such parallelisms, suggesting that they see some underlying similarity despite the differences in terms of lexical units or locality constraints. Because we think that the pursuit of these parallelisms have led to substantial progress (see Patel, 2008 in the domain of music), we don't see why the domain of action should be treated differently.

Second, we think that Moro's classical stance is counterproductive on evolutionary grounds. In fact, it suffers from the same limitations that Gary Marcus points out for classical modularity (Marcus, 2006). Marcus argues forcefully, and persuasively, for the need for a serious injection of Darwinian thinking in the domain of cognitive science, and we would add, especially in the context of generative grammar. Specifically, we think that in his arguments Moro underestimates the benefits of Darwin's great idea of descent with modification. “Descent” thinking is key to construct cognitive phylogenies, and several authors have provided experimental arguments in favor of a relation between syntax and action grammar in this context (Stout, 2010; Uomini and Meyer, 2013). But as Darwin knew well, descent is often accompanied by modification. Hence we expect differences across related domains. Such differences may in fact obscure deep similarities (cf. the vibrant area of research on deep homologies in current biology). We think that at this point, focusing on the similarities between syntax and action grammar could lead productive work on neurolinguistics, along the lines argued for by Pulvermüller (see Roy et al., 2013 for work on specific language impairment).

In addition, we think that both Moro and Pulvermüller may not be focusing on the most productive mode of comparing syntax and action. Both authors focus on the hierarchical structures in both domains, but we contend that it may be more productive to focus on the generative operation underlying these structures [what Moro refers to as Merge, although we want to keep an open mind about what the relevant elementary computational mechanism(s) may be]. Breaking down the relevant domains into primitive operations would enable the (we think, very real) possibility that the same underlying mechanism is deployed in a variety of cognitive domains, with differences arising from the ways in which these domains are embedded within larger cognitive systems. (Such differences may well be the causes of numerous dissociation effects reported in the modularity literature.) For instance, the fact that natural language syntax manipulates units with conceptual content may account for certain differences between, say, language and music, even if the core combinatorial operation is shared by the two systems (Roberts, 2012). Other properties of operations, such as binarity (giving rise to binary branching structures), may be relaxed in domains like arithmetic (Chomsky, 2008) or vision (Jackendoff, 2007), and may therefore be best thought of as domain-specific constraints on the same operation imposed by different interfacing systems.

Finally, as linguists, we would like to point out that the two arguments offered by Moro against a deep relation between syntax and action grammar are somewhat obsolete in the context of current linguistic theorizing. Much current work in syntax has come to view “words” (the atoms of computation that Moro takes to make syntax different) as the output of the syntactic computation, not its input (Halle and Marantz, 1993, and much work in the framework of Distributed Morphology). As for locality conditions, here too syntacticians have come to the conclusion that these may be best seen as constraints acting as post-syntactic filters (see Boeckx, 2012 for a recent survey). So both “words” and “locality” effects may not be so intrinsic properties of natural language syntax (or better said, its core computational process), and should therefore not stand in the way of possible relations across mental capacities of our species.

## Conflict of interest statement

The authors declare that the research was conducted in the absence of any commercial or financial relationships that could be construed as a potential conflict of interest.
